# Persimmon Leaves: Nutritional, Pharmaceutical, and Industrial Potential—A Review

**DOI:** 10.3390/plants12040937

**Published:** 2023-02-18

**Authors:** Abul Hossain, Fereidoon Shahidi

**Affiliations:** Department of Biochemistry, Memorial University of Newfoundland, St. John’s, NL A1C 5S7, Canada

**Keywords:** persimmon leaf, *Diospyros kaki* L., flavonoids, terpenoids, health benefits, seasonality, geographical locations

## Abstract

Persimmon is a delicious fruit, and its leaves are considered a valuable ingredient in food, beverage, pharmaceutical, and cosmetic sectors. Traditionally, persimmon leaves (PL) are used as a functional tea in Asian culture to cure different ailments, and are also incorporated into various food and cosmeceutical products as a functional ingredient. PL mainly contain flavonoids, terpenoids, and polysaccharides, along with other constituents such as carotenoids, organic acids, chlorophylls, vitamin C, and minerals. The major phenolic compounds in PL are proanthocyanidins, quercetin, isoquercetin, catechin, flavonol glucosides, and kaempferol. Meanwhile, ursolic acid, rotungenic acid, barbinervic acid, and uvaol are the principal terpenoids. These compounds demonstrate a wide range of pharmacological activities, including antioxidant, anticancer, antihypertensive, antidiabetic, anti-obesity, anti-tyrosinase, antiallergic, and antiglaucoma properties. This review summarizes the latest information on PL, mainly distribution, traditional uses, industrial potential, and bioactive compounds, as well as their potential action mechanisms in exhibiting biological activities. In addition, the effect of seasonality and geographical locations on the content and function of these biomolecules are discussed.

## 1. Introduction

Persimmon, mainly *Diospyros kaki* L., is primarily cultivated in Eastern Asia, including China, Japan, and Korea, and is also grown in India, Azerbaijan, Spain, Türkiye, Brazil, and the USA. Persimmon fruit is eaten fresh or dried (primarily astringent type), while leaves are well-known as functional tea in East Asian countries. In Japanese culture, PL are infused with hot water and consumed as functional tea (“kakinoha-cha”), which is traditionally known for curing paralysis, burns, and frostbite, as well as to stopping bleeding [1,2]. Persimmon trees are deciduous, hence their leaves are of interest as functional food ingredients, nutraceuticals, and pharmaceuticals. Persimmon leaves (PL) contain a wide range of bioactive compounds such as phenolics (flavonoids), terpenoids, polysaccharides (cellulose, hemicelluloses, and lignins), caffeine, carotenoids (kryptoxanthin), amino acids, vitamin C, minerals, and chlorophylls. In particular, the leaves are abundant in phenolic compounds, including proanthocyanidins, quercetin, isoquercetin, catechin, flavonol glucosides, myricitrin, and kaempferol, thus exhibiting antioxidant, anticancer, antiallergic, anti-inflammatory, antidiabetic, antihypertensive, and vasorelaxant effects [3,4]. For example, Huang et al. [5] identified 32 compounds in PL using high-performance liquid chromatography–quadrupole time of flight–mass spectrometry (HPLC-QTOF-MS) in negative ion mode, and the major compounds were hyperoside, quercetin, kaempferol, myricetin, trifolin, vitexin, astragalin, 19α,24-dihydroxy ursolic acid, barbinervic acid, and pomolic acid.

PL have attracted much attention in the cosmeceutical industries in Asian countries due to their antioxidant, anti-tyrosinase, anti-inflammatory, photo-protective, antiallergic, antibacterial, and antiwrinkle effects [6]. Moreover, PL have shown positive effects against atherosclerosis, osteoporosis, hemostasis, apoplexy, constipation, hypertension, and neurodegenerative diseases, such as Parkinson’s and Alzheimer’s diseases [7,8,9]. In particular, these leaves are considered as folk medicine in Asian cultures and are used as dietary supplements. For example, Naoxinqing (NXQ) tablets prepared from the ethyl acetate extract, mainly flavonoids (over 25%), of PL have been used as traditional Chinese medicine to prevent and cure neurodegenerative diseases [5]. In addition, animal studies found that the leaves were not toxic [10]. Therefore, this review attempts to summarize the up-to-date research status of PL and their bioactive compounds, along with their functions. It also discusses the nutritional and pharmacological properties along with various chemical structures and their relationship in demonstrating beneficial effects in food preservation and health promotion. In addition, the industrial potential of these leaves for use as functional ingredients in food, pharmaceutical, and cosmeceutical industries is provided.

## 2. Distribution of PL

Persimmon is broadly cultivated in the subtropical and tropical regions of East Asia. Oriental persimmons have been cultivated commonly in China, Japan, and Korea and have been the fifth fastest-developing fruit in the world. The world production of persimmon totaled around 5 million tons (0.75% of global fruit production), where China supplies most of the world’s total fruits [11,12]. Apart from Chinese cultivars (e.g., Hachiya, Tamopan, Fuyu, Tanenashe, Ormond, and Imoto), Korea (e.g., Hongosi, Hachiya, Dan Gam/Fuyu), Spain (Homan red), Japan (e.g., Hachiya, Taubata, Tamopan, Tanenashe, Fuyu, Imoto, Suruga, and Jiro), Brazil (e.g., Sibugaki, Hatemya, Hachiya, and Trakoukaki), Azerbaijan, Uzbekistan, Italy, and New Zealand are the other major persimmon producing countries [6].

**Figure 1:**
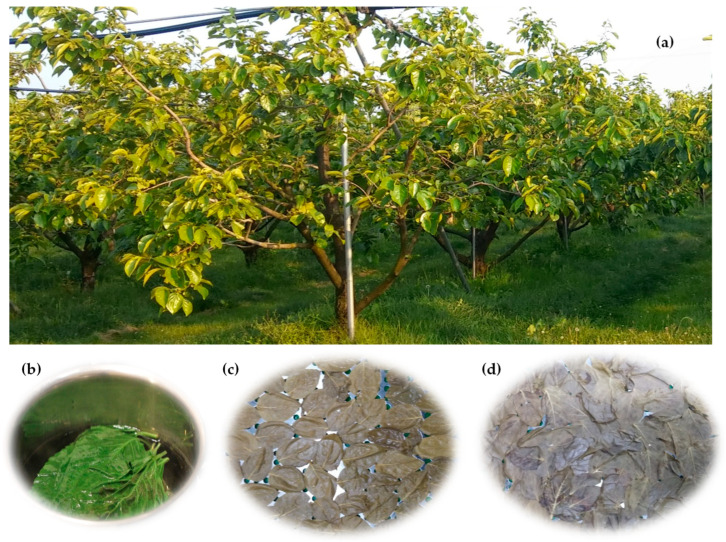
Persimmon (*D. kaki*) plant (**a**), and the preparation of tea (**b**), blanched leaves (**c**), and dried leaves (**d**).

The most widely cultivated species of persimmon is oriental persimmon (*Diospyros kaki* L.), which belongs to the family of Ebenaceae. Moreover, date-plum (*Diospyros lotus*) and velvet-apple (*Diospyros discolor*) are native to southeast Europe and southwest Asia, whereas American persimmon (*Diospyros virginiana*) and Texas persimmon (*Diospyros texana*) are native to the United States. Generally, the trees of persimmon can reach 4.5–18 m tall, and their leaves are 7–15 cm long, with brown-hairy petioles (Figure 1).

These leaves are deciduous and bluish-green in color, which turn into orange, yellow, or red in the autumn. They are simple, alternate, and ovate elliptic in shape and have a mild flavor with a slightly bitter, astringent, and sweet taste. The fruit of this plant are typically harvested during the fall season (September to October) [10]. Almost every part of the persimmon plant, including fruits, peels, seeds, leaves, and bark, has been used in traditional medicine, especially young leaves, which are mainly used as functional tea due to their abundance of antioxidants. Generally, mature leaves with less antioxidative compounds are not considered for tea, and this could be due to the movement of bioactive compounds (e.g., antioxidants) from leaves to fruits during fruiting [13].

## 3. Traditional Uses and Industrial Potential

Young PL have long been used as a traditional tea (gamiph-cha, kakinoha cha, or guilin ye qing tea) in Asian cultures. This is a caffeine-free tea, which tastes slightly bitter with a very pleasant flavor, consumed mainly to promote a healthy diet and as an anti-aging ingredient due to the presence of high levels of flavonoids and vitamin C [14,15]. Tea made from dried leaves is very common, but it is also prepared from fresh green leaves. The major compounds of PL are flavonoids, tannins, vitamins, and terpenoids. PL have long been considered a traditional medicine to treat ischemic angina, stroke, internal hemorrhage, hypertension, atherosclerosis, and infectious diseases, as well as paralysis, frostbite, burns, and constipation, which have been mentioned in ancient Chinese medical books [10,16]. In particular, NXQ is a protected drug of Traditional Chinese Medicine and is used in managing cardio- and cerebrovascular diseases [5]. Traditionally, PL have also been used to promote maternal health [10]. Furthermore, NXQ has been shown to have beneficial effects for the treatment of atherosclerosis, stroke, transitory ischemia syndrome, cerebral thrombosis sequela, cerebral thrombogenesis, cerebral embolism, and apoplexy sequela, among others [17]. Bei et al. [18] found that NXQ shields NG108-15 cells against H_2_O_2_-induced oxidation by inhibiting apoptosis and improving redox disequilibrium. In addition, the extracts of the leaves are considered as food additives and are incorporated into beverages, candies, biscuits, sushi, noodles, and rice cakes to improve shelf life and functional properties [4,19]. So far, various persimmon leaf-based products, such as persimmon leaf tea (good for eye health), herbal tea, caffeine-free tea (Hankook), dried leaves, powder, herbal oil, glycerite liquid extract, capsules, and soap, have been launched in the marketplace, including by Amazon.com, Inc., and their price can reach up to USD 200/oz.

In Japan, PL have traditionally been used for tempura making and sushi wrapping due to their antimicrobial and health-promoting effects. In addition, PL may be incorporated in athlete’s foot socks and soaps as well as in petrochemical materials, especially those related to textile industries and military equipment [13,20]. Furthermore, it is believed that having PL tea in the daytime and washing the body or face with these leaves would ultimately result in skin whitening [10]. On the other hand, PL have been reported to show an inhibitory effect against steel corrosion, which could be utilized for industrial applications. For example, Gerengi et al. [21] reported the inhibitory effect of PL extract on steel corrosion in a HCl solution. It was found that the PL extract was an excellent inhibitor (up to 91% inhibition) for St37 steel, which was measured using chemical, electrochemical (electrochemical impedance spectroscopy (EIS), potentiodynamic polarization (PDP), and dynamic electrochemical impedance spectroscopy (DEIS)), as well as surface morphological screening (energy dispersive spectroscopy (EDS), scanning electron microscopy (SEM), and Fourier transform infrared spectroscopy (FTIR)) techniques. Additionally, Go and Song [22] prepared packaging films using green algae and PL and found that the incorporation of PL (1%) into the packaging materials improved film flexibility and antioxidant activity, and this could be due to the abundance of phenolic compounds in PL.

## 4. Proximate Composition of PL

PL are commonly used in a dried form to make functional tea. The moisture content of fresh persimmon is around 75–80%, while the powder obtained from dried leaves contains 10 and 4% moisture when dried using hot-air and freeze-drying, respectively [1]. The major pigments in PL are chlorophylls, xanthophylls, anthocyanins, and carotenes, which are responsible for changing colors throughout the growing season. Hossain et al. [1] investigated the Hunter color values of the most common PL grown in Korea and found that L* (lightness), a* (yellowness), and b* (redness) values were 57.92–62.14, 1.42–2.31, and 15.96–20.13, respectively, for the freeze-dried leaves. In contrast, Chung et al. [19] found that the L*, a*, and b* values were 43.55, −15.05, and 26.83, respectively, for fresh PL. The same study also reported that the contents of chlorophyll *a* and *b* of the fresh PL were 711.13 and 211.97 μg/g, respectively. Contrarily, the contents of chlorophyll *a* and *b* of the Korean PL tea were 0.22 and 0.52 mg/g, respectively [23]. Furthermore, Hassan et al. [24] reported that freeze-dried PL (*D. lotus*) harvested from Korea contained 12.6–14.6 mg/g of vitamin C. Similarly, the content of vitamin C in freeze-dried PL (*D. Kaki*) obtained from various regions of Korea during the flowering stage was 7.53–14.87 mg/g [1]. It was also reported that the PL were a good source of minerals, mainly K (538.24–971.58 mg/100 g), Ca (255.7–591.25 mg/100 g), Mg (69.07–153.33 mg/100 g), Mn (4.78–36.49 mg/100 g), Fe (3.44–13.77), Na (7.05–12.63), Cu (1.54–5.8), and Zn (0.76–2.34). Furthermore, the major sugars in PL were sucrose, fructose, glucose, maltose, and xylose, where sucrose and fructose contributed more than 70% of the total sugar content [19,25]. The major fatty acids in PL are myristic, palmitic, stearic, 10-octadecenoic, cerotic, linoleic, and linolenic acids [10].

## 5. Bioactive Compounds of PL

PL contain a high amount of flavonoids (e.g., astragalin, hyperin, isoquercitrin, kaempferol, and quercetin) and terpenoids along with other compounds, including chlorophylls, carotenes, kryptoxanthin, cellulose, hemicelluloses, and lignins. These compounds exhibit potential antioxidant, antihypertensive, anti-inflammatory, anticancer, antidiabetic, antiallergic, and antimicrobial effects. The major compounds found in PL are described below.

### 5.1. Phenolic Compounds

Phenolic compounds are secondary metabolites containing hydroxylated aromatic rings, which play an essential role in the growth and development with a defense mechanism in the plant. In particular, they engage in plants’ defense by inhibiting herbivory, ultraviolet radiation, and pathogen attacks. In addition, they are responsible for the flavor, color, bitterness, and astringency of fruits such as persimmon [26]. Phenolic compounds in plants primarily originate from phenylalanine, and to a lesser extent, tyrosine. For example, *p*-hydroxycinnamic acid is derived from tyrosine with the assistance of tyrosine ammonia-lyase (TAL), whereas *trans* cinnamic acid is derived from phenylalanine, catalyzing by phenylalanine ammonia-lyase (PAL). The major phenolics present in plants are flavonoids, phenolic acids, tannins, stilbenes, lignans, and coumarin [26,27,28]. Phenolic compounds are well known as antioxidants, which demonstrate inhibitory activities against α-glucosidase and tyrosinase activities as well as LDL-cholesterol, DNA, and lipid oxidation [29].

**Figure 2:**
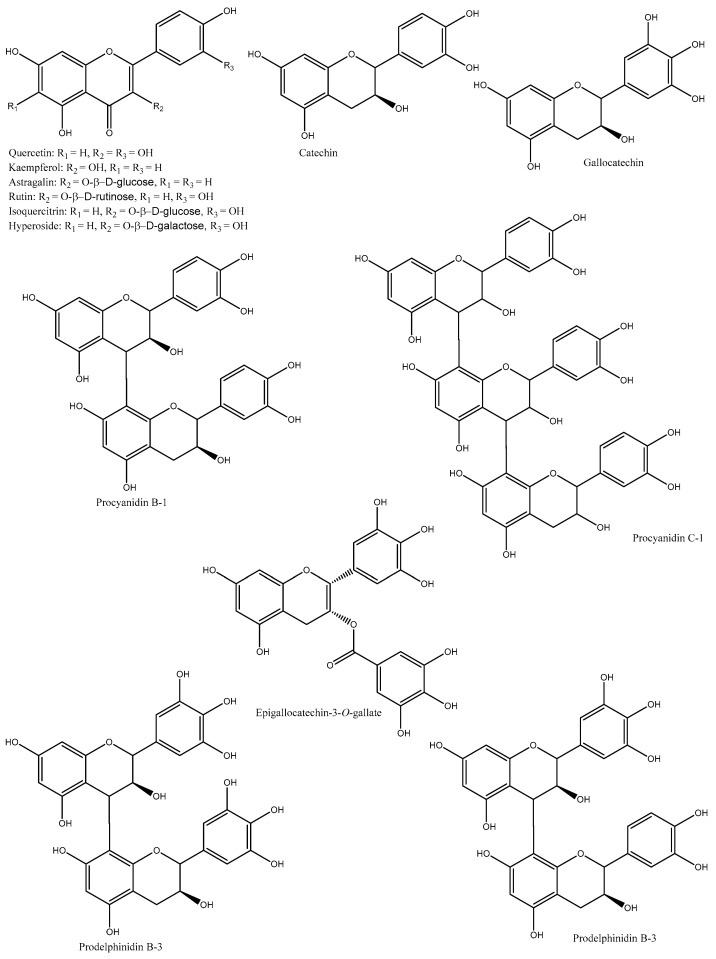

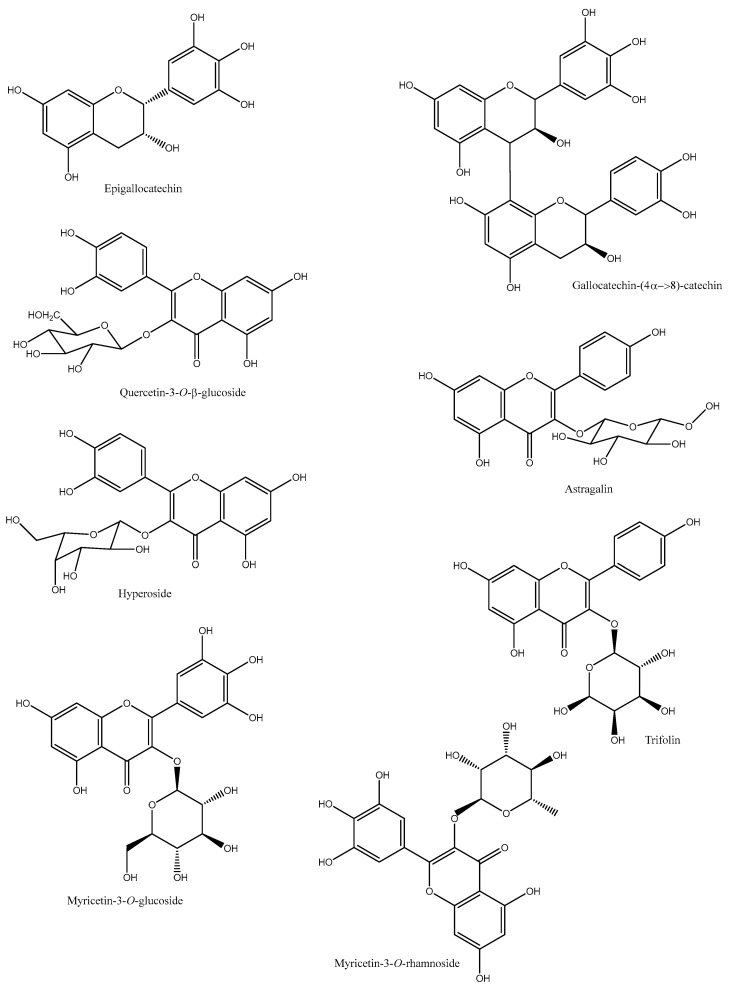
Major phenolic compounds found in PL.

Phenolics in PL are mainly flavonoids, including flavonol (e.g., quercetin, isoquercetin, kaempferol, and myricetin) and flavonol glucoside (e.g., quercetin-3-*O*-β-L-arabinopyranoside, quercetin-3-*O*-β-D-glucopyranoside, quercetin-3-*O*-β-D-galactopyranoside, kaempferol-3-*O*-α-L-rhamnopyranoside, kaempferol-3-*O*-β-D-galactopyranoside, kaempferol-3-β-D-xylopyranoside, kaempefrol-3-*O*–L-arabinopyranoside, kaempferol-3-*O*-(2″-*O*-galloyl)-β-D-glucopyranoside, myricetin-3-*O*-α-D-glucopyranoside, and quercetin-3-*O*-β-D-galaetoside) [10] (Table 1 and Figure 2).

For example, Choi et al. [32] identified four flavonoids from PL, namely isoquerercitrin, quercetin 3-*O*-β-D-glucopyranoside-2″-gallate, kaempferol 3-*O*-β-D-glucopyranoside-2″-gallate, and astragalin. Similarly, nine flavan-3-ols were isolated from PL, mainly catechin, gallocatechin, and pyrocyanidins [30]. Moreover, Kawakami et al. [33] reported that the major phenolics in PL were unique proanthocyanidin oligomers, such as catechin, epicatechin, epigallocatechin-3-*O*-gallate, epigallocatechin, epicatechin-3-*O*-gallate, and prodelphinidin. Likewise, Tao et al. [38] suggested that proanthocyanidins of PL were mainly catechin with a B-type link along with a small portion of catechin gallate, gallocatechin, and an A-type link. Finally, Cho et al. [39] stated that the 60% ethanol was the best solvent to extract phenolics, which were mainly composed of (+)-gallocatechin and prodelphinidin B-3.

Bei et al. [40] identified four flavonoids (quercetin, kaempferol, hyperin, and astragalin) from PL using HPLC. An aqueous extract was prepared from the Korean PL, which contained quercetin 3-*O*-2′galloylglucoside and kaempferol 3-*O*-2′galloylglucoside [41]. The compositions and contents of phenolics were investigated from eight varieties of PL harvested in Taiwan [36]. The major compounds were flavonoid, condensed tannin, and phenolic acids, including kaempferol-3-*O*-rhamnoside, myricetin, naringin, quercetin-3-*O*-rhamnoside, quercetin-3-*O*-glucoside, quercetin-3-*O*-galactoside, sinapic acid, gallic acid, and *p*-hydroxybenzoic acid, among others. Heras et al. [2] dried PL using various drying techniques (hot-air-drying at 100 and 180 °C, shade-drying, and freeze-drying) and extracted phenolic compounds by aqueous extraction (70, 80, and 90 °C for 1, 3, 5, 60, and 1440 min). It was found that PL dried under air-drying at 100 °C and extracted at 90 °C for 60 min provided the optimal process for the extraction of phenolic compounds. In another study, Heras et al. [3] identified and quantified 41 phenolic compounds from PL using liquid chromatography (LC) coupled with mass spectrometry (MS); the major compounds were simple phenolic acids, hydroxycinnamic acids, hydroxybenzoic acids, flavanols, flavanones, flavonols, flavonechalcones, tyrosols, and their conjugated derivatives. So far, this is the highest number of phenolic compounds identified from PL. Meanwhile, a new flavonoid (kaempferol-3-*O*-β-D-2″-coumaroylgalactoside) along with kaempferol-3-*O*-β-D-2″-feruloylglucoside were identified from the Korean PL [16]. The overall identified chemical compounds were 14 flavonoids, 7 triterpenoids, 2 coumarins, 1 ionone, and 1 acetophenone. Furthermore, the major phenolic compounds of six selected persimmon cultivars in Japan were investigated using a reverse-phase HPLC, and their structures were confirmed by NMR [34]. The identified compounds were isoquercitrin, hyperoside, trifolin, chrysontemin, astragalin, kaempferol-3-*O*-(2″-*O*-galloyl-β-D-glucopyranoside), and quercetin-3-*O*-(2″-*O*-galloyl-β-D-glucopyranoside).

NXQ prepared from PL is used for the management of cardio- and cerebrovascular diseases, and its composition was investigated using UPLC coupled with a tandem MS [42]. Seven compounds, specifically kaempferol-3-*O*-glucoside (astragalin), quercetin-3-*O*-glucoside (isoquercitin), quercetin-3-*O*-galactoside (hypericin), kaempferol, quercetin, pyromucic acid, and protocatechuic acid were identified in the NXQ. Furthermore, Wang et al. [43] isolated a novel compound named vomifoliol 9-*O*-α-arabinofuranosyl (1→6)-β-D-glucopyranoside. However, most of these studies investigated the soluble phenolics of PL though insoluble-bound phenolics (IBPs) are abundant in leaves (up to 70%); thus, attention should be paid to the extraction of IBPs from PL in order to fulfill the overall phenolic profile [44].

### 5.2. Terpenoids

**Figure 3:**
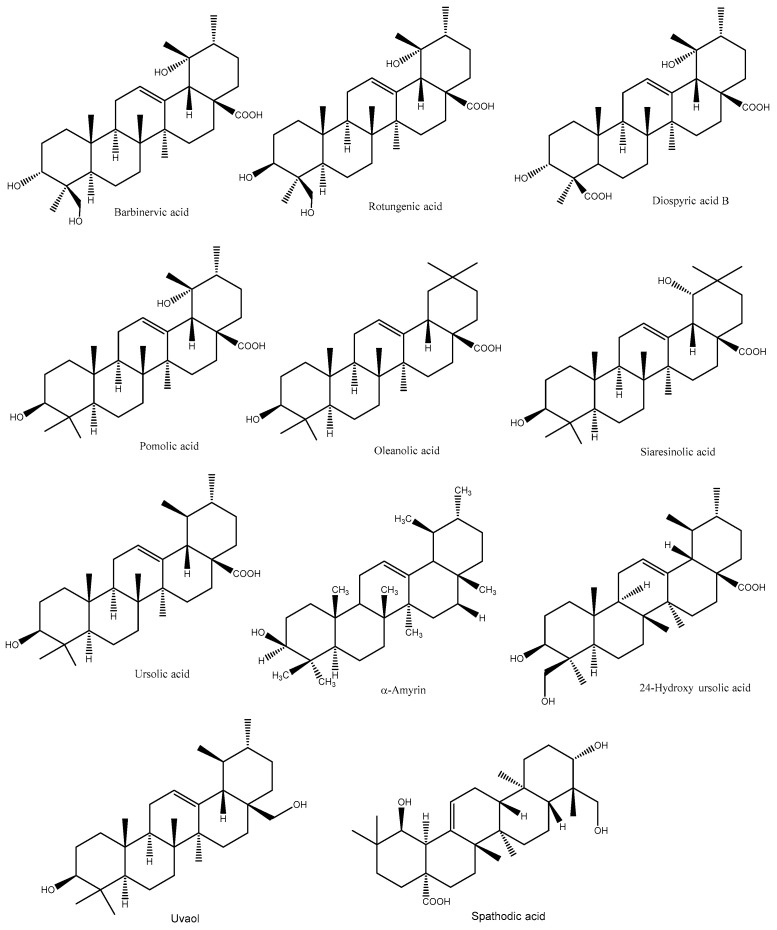
Major triterpenoids in PL.

Terpenoids are a large class of diverse organic compounds, occurring naturally in plants to protect against biotic and abiotic stresses. Based on the number of isoprene units, terpenoids can be classified into monoterpenes (10 carbons), sesquiterpenes (15 carbons), diterpenes (20 carbons), sesterpenes (25 carbons), and triterpenes (30–40 carbons) [45]. They are the major constituents of essential oils (EOs) and exhibit several pharmacological and biological activities [45]. The major terpenoids found in PL are mainly pentacyclic triterpenoids such as ursolic acid, 19,24-dihydroxyursolic acid, 19-hydroxyursolic acid, 24-hydroxyursolic acid, siaresinolic acid, oleanolic acid, barbinervic acid, rotungenic acid, amyrin, and uvaol, among others (Figure 3).

For instance, Fan and He [46] developed an HPLC method to identify triterpene acids from PL, and the compounds were rotungenic acid, 24-hydroxyursolic acid, and barbinervic acid and its epimer. Similar to this study, seven triterpenoids were isolated from the Korean PL, namely barbinervic acid, diospyric acid B, pomolic acid, rotungenic acid, oleanolic acid, siaresinolic acid, and ursolic acid [16]. Moreover, Chen et al. [47] reported five triterpenoids such as ursolic acid, α-amyrin, uvaol, 19 α,24-dihydroxyursolic acid, and 19α-hydroxy ursolic acid in PL. Similarly, three minor novel triterpenoids and a known terpenoid (rosamultin) were identified from Chinese PL using NMR [48]. The novel triterpenoids were kakisaponin B (28-*O*-β-D-glucopyranosyl-3α,19,24-trihydoxy-18,19-secours-11,13(18)-dien-28-oic acid), kakisaponin C (28-*O*-β-D-glucopyranosyl-2α,3α,19-trihydoxy-18,19-secours-11,13(18)-dien-28-oic acid), and kakidiol (C_29_-triterpene with an aromatic E-ring structure). In another study, they identified another novel triterpene compound (18,19-*seco*-3β-hydroxy-urs-12-en-18-one) along with five known compounds (uvaol, oleanolic acid, ursolic acid, (−)-syringaresinol, and (−)-syringaresinol-4-β-D-glucopyranoside) [49]. In addition, two new ursane-type triterpenoids, namely 3α,19α-dihydroxyurs-12,20(30)-dien-24,28-dioic acid and 3α,19α-dihydroxyurs-12-en-24,28-dioic acid, along with 12 known ursane- and oleanane-type triterpenoids (coussaric acid, rotungenic acid, barbinervic acid, pomolic acid, ursolic acid, oleanolic acid, 24-hydroxyursolic acid, 24-hydroxy-3-*epi*-oleanolic acid, 24-hydroxy-3-*epi*-ursolic acid, 19,24-dihydroxyurs-12-en-3-on-28-oic acid, and spathodic acid) were identified from the Korean PL [50].

### 5.3. Polysaccharides

Polysaccharides of PL are mainly a group of hetero-polysaccharides, and the most common polysaccharides units are glucose, galactose, arabinose, mannose, and rhamnose. For example, Park et al. [51] suggested that polysaccharide fraction I of PL is mainly composed of galactose (29.9%), galacturonic acid (16.7%), arabinose (17.8%), rhamnose (10.4%), and trace amounts of 3-deoxy-D-manno-2-octulosonic acid (KDO)-like materials (0.9%). Fraction II was composed of 27.2% acidic sugars (glucuronic acid and galacturnonic acid), 19.6% arabinose, 19.4% rhamnose, 13.6% galactose, and 9.6% KDO-like compounds, including 2-methyxylose (3.3%), 2-methylfucose (3.0%), and KDO (3.1%). Moreover, fraction III consisted of acidic sugars (31.4%), arabinose (14.6%), rhamnose (15.9%), galactose (12.6%), and KDO-like materials (1.7%). In another study, Park et al. [11] reported that the polysaccharides of PL mainly consisted of arabinose (20%) and galactose (17.9%) along with uronic acids (glucuronic and galacturonic acids) and unusual sugars, such as 2-*O*-methylxylose, 2-*O*-methylfucose, apiose, KDO, and 3-deoxy-D-lyxo-2-heptulosaric (DHA). Furthermore, a few studies have reported that the polysaccharide composition of PL was mainly neutral sugars (58.1–78.6%), uronic acids (26.2–38.3%), and KDO-like materials (2.5–4.43%); the major sugars being fucose, 2-methylfucose, 2-methylxylose, rhamnose, arabinose, galactose, glucose, mannose, xylose, apiose, galacturonic acid, and glucuronic acid [12,52,53,54].

### 5.4. Other Compounds

**Figure 4:**
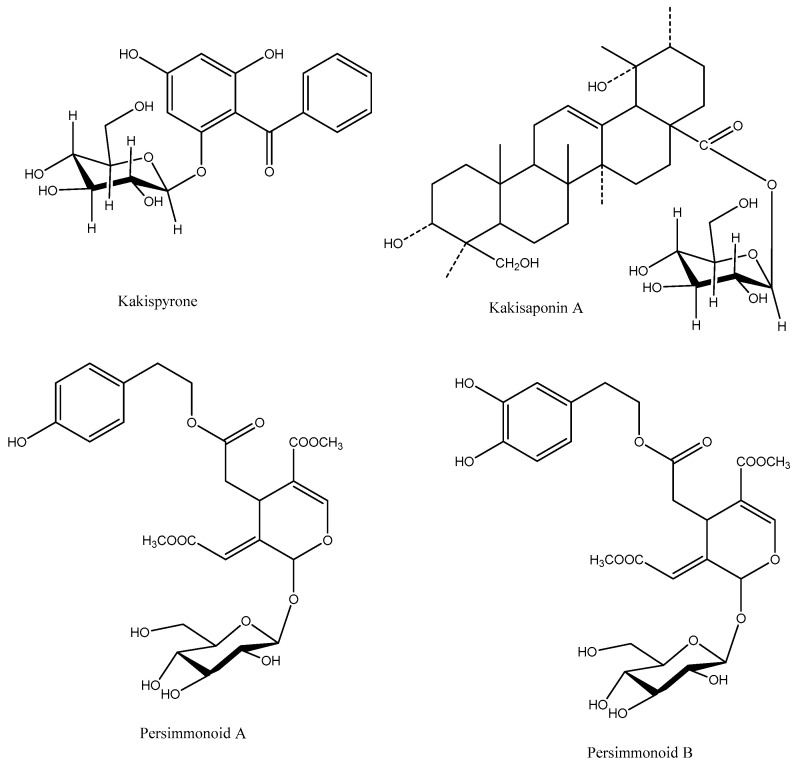
Chemical structures of kakispyrone, kakisaponin A, and persimmonoid A and B.

Other compounds such as naphthoquinones (3-bromoplumbagin, 3-methoxy-7-methyluglone, 8′-hydroxy-isodiospyrin, martinone, isodiospyrin, diospyrin, neodiospyrin, mamegakinone, and 7-methyluglone), organic acids (benzoic, succinic, salicylic, pyromucic, indoleacetic, and procatechuic acids), and coumarins (6–7-hydroxyl-7-hydroxycoumarin and scopoletin) have been reported in PL [10]. Secoiridoids and lignans were identified from PL, which were mainly persimmonoid A and B, ligustroside, oleuropein, medioresinol, syringaresinol, pinoresinol, medioresinol monoglucoside, syringaresinol-β-D-glucoside, and pinoresinol-β-D-glucoside, isolariciresinol [55]. Additionally, Chen et al. [56] identified two new compounds, such as 4,6-dihydroxy-2-*O*-β-D-glucopyranosylbenzophenone (kakispyrone) and kakisaponin A, from PL along with 11 known compounds, mainly phenolic compounds (Figure 4).

## 6. Effect of Seasonality and Geographical Location on the Chemical Composition of PL

Geographical location and harvesting times play a vital role in the chemical composition of PL. For example, Korean PL were harvested at three different maturity stages, including early June (young stage), early August (early green mature stage), and early October (full mature stage), and it was found that seasonality had a great impact on the sugar compositions and glycosidic linkages in the polysaccharides [12]. It was found that the yield of polysaccharides decreased with increasing harvesting times. However, samples collected during early August were composed of higher amounts of arabinose, galactose, xylose, rhamnose, and galacturonic acid, exhibiting the presence of β-glucopyranoside linkages. Kim and Lee [57] suggested that the contents of fat, amino acids, fiber, vitamin C, and ash of Korean PL increased during growth and started to decrease after mid-June, while the content of moisture decreased during the growing season. Moreover, Clark and Smith [58] investigated the content of macro- and micronutrients in the youngest-mature-PL harvested from New Zealand during fruiting and non-fruiting stages over two seasons. Concentrations of most of the elements were lower in the early growing season, followed by increasing mid-season, and started declining again at the end of the season. In another study, Hossain et al. [1] suggested that the concentration of Ca, Mg, K, and Mn increased from May to June but decreased for Fe, Na, Cu, and Zn in PL collected from Korea. Moreover, PL harvested in May (flowering stage) had a higher content of vitamin C and moisture than those harvested in late June (fruiting stage). This could be due to the movement of these constituents from the leaves to the fruits at the fruiting stage. In addition, plant physiological characteristics, environmental conditions, light intensity, and the level of minerals (e.g., nitrogen) present in the soil could play a role in decreasing these constituents during fruiting [1].

Seasonality, geographic location, and growing conditions also have a great effect on the content and function of secondary metabolites of PL. For example, Chang et al. [36] harvested PL from eight persimmon varieties in different periods (September to November) from Taiwan. PL collected in the period with higher solar radiation, temperature, and sunshine duration had higher phenolic contents (total polyphenols, flavonoids, condensed tannins, and phenolic acids) and antioxidant activity. The content of phenolics was higher in September, followed by October and November in the individual phenolic compounds, mainly gallic acid, *p*-hydroxybenzoic acid, sinapic acid, quercetin-3-*O*-rhamnoside, and myricetin. Moreover, the contents of total phenolics, flavonoids, and tannins, as well as their antioxidant activities were higher in the flowering stage (late May) than in the fruiting stage (late June) in the Korean PL [13]. Similarly, Hossain et al. [4] harvested PL from five Korean cultivars during the flowering and fruiting stages, and then dried them using hot-air-drying and freeze-drying. It was found that PL, mainly ‘Gabjubaekmok’, collected during the flowering stage and dried by hot air, was richer in phenolic compounds. Likewise, Jeong et al. [59] suggested that the contents of phenolics and flavonoids of Korean PL were increased in a time-dependent manner, and in June, they reached in their peaks. In another study, Korean PL were harvested from May (early stage) to November (mature stage) and it was found that the PL obtained in May had higher levels of total phenolics, flavonoids, and antioxidant activity compared to other months [60]. Furthermore, the content of phenolics in PL harvested from Japan reached a maximum value in June among 11 different growing stages (April to November) and then gradually decreased throughout the season [33]. Kawakami et al. [61] found changes in flavonol glycosides in PL harvested from April to October in Japan, where flavonol glycosides increased until June, and then they were stable during later growth stages. In particular, four non-galloylated flavonol glycosides were identified at the leaf-shooting stage in April, and in early May, fouradditional galloylated flavonol glycosides began to accumulate, resulting in eight flavonols by June, which could be detected in the mature PL until autumn. Furthermore, Fan et al. [46] stated that the content of triterpene acids (barbinervic, rotungenic, and 24-hydroxyursolic acids) varied significantly in the PL growing in different locations in China. In addition, the major flavor compounds in Korean PL were alcohols (*cis*-3-hexenol, linalool, and 1-α-terpineol) and aldehydes (nonanal, *trans*-2-hexanal, and 2-decenal), and they were highest in the leaves harvested in May rather than in June and July [62].

## 7. Pharmacological Effects of PL

**Figure 5:**
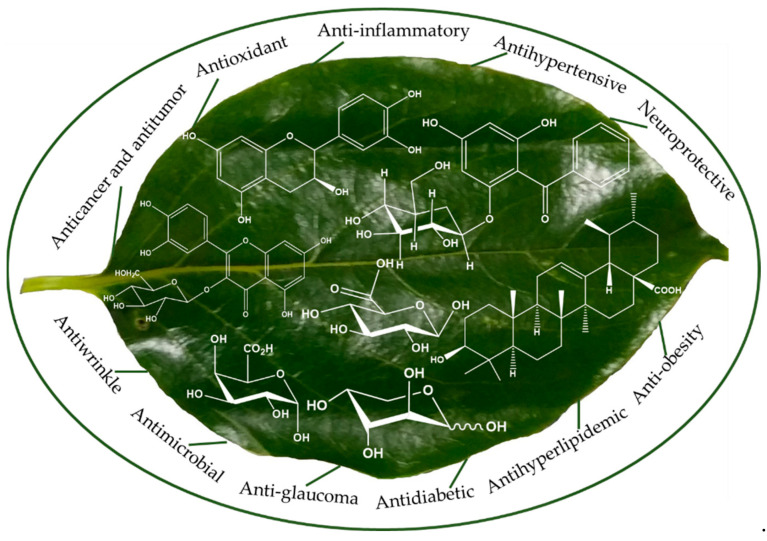
Pharmacological properties of PL.

Traditionally, PL are used for curing ischemic stroke, hypertension, atherosclerosis, paralysis, frostbite, burns, and constipation, among others. However, numerous in vitro and in vivo studies have assessed the potential health benefits, including antioxidant, anti-atherosclerosis, antidiabetic, antihyperlipidemic and anti-obesity, anticancer and antitumor, anti-glaucoma, antihypertensive, anti-inflammatory, immunostimulatory, neuroprotective, antiallergic and antiwrinkle, and antimicrobial properties, of PL (Figure 5). A summary of biological and medicinal effects of PL is provided in Table 2.

### 7.1. Antioxidant Effects

Persimmon leaves have been extensively studied for their antioxidant activities using in vitro assays (Table 3).

PL extracts, rich in flavonoids, possessed strong free radical scavenging activity. Choi et al. [32] identified four flavonoids from PL and reported their strong antioxidant activity compared to α-tocopherol. Quercetin 3-*O*-β-D-glucopyranoside-2″-gallate especially, was the most prominent flavonoid with inhibitory activity against lipid peroxidation. Likewise, antioxidant activities (DPPH radical scavenging, ferric reducing power, TEAC, and inhibition copper-induced human LDL oxidation) of eight PL varieties harvested from Taiwan at various harvesting times (September to November) were investigated [36]. Results suggested that harvest time and variety were important factors for antioxidant activity, mainly those harvested in months with higher ambient temperature, sunshine duration, and solar radiation. Moreover, Kazzem et al. [42] determined the antioxidant activity of NXQ tablets prepared from PL. It was found that flavonoids, mainly quercetin and kaempferol, showed strong DPPH radical scavenging activity and inhibited the H_2_O_2_-induced human vascular endothelial (EA.hy926) cell injury and intracellular ROS generation. Likewise, flavonoids and terpenoids were identified from the Korean PL and it was found that kaempferol and quercetin derivatives, mainly those with galloyl moieties, exhibited strong antioxidant activities using DPPH and HPLC-ABTS assays [16]. Furthermore, water-soluble PL extracts showed strong antioxidant activities using DPPH, ABTS, nitric oxide (NO), and reducing power assays [68], whereas methanolic extracts were able to decrease the production of malondialdehyde (MDA) [84]. Similarly, Li et al. [75] stated that flavonoids of PL decreased MDA production and increased superoxide dismutase (SOD) activity in hyperlipidemic rats. In a similar study, Sun et al. [71] reported that flavonoids of PL showed significantly higher scavenging activity of hydroxyl radicals and superoxide anions, reducing power, and metal chelating activity than that of rutin (standard compound). In addition, they also suggested that PL significantly reduced the level of ROS and MDA while improving the activity of SOD, catalase, and glutathione peroxidase (GSH-Px) in MC3T3-E1 cells in a dose-dependent manner, suggesting the antioxidant potential of PL. In another study, flavonoids of PL showed the potential to attenuate H_2_O_2_-induced apoptosis in MC3T3-E1 cells via the nuclear factor kappa B (NF-kB) pathway [9]. Furthermore, Yoo et al. [80] reported the scavenging activity of PL extracts against DPPH and superoxide anion radicals, as well as inhibitory activity against 5-lipoxygenase (5-LO) and cyclooxygenase (COX). Furthermore, the antioxidant activities of Japanese PL tea that were measured using β-carotene bleaching and hydroxyl radical scavenging assays were higher than or equal to that of ascorbic acid, while superoxide anions and DPPH radicals were also greatly scavenged by the leaves [15]. Moreover, Ashry et al. [85] claimed that PL could reduce radiation-induced oxidative stress and tissue injury in irradiated rats due to its antioxidant nature, specifically, its free radical-scavenging ability. This is because irradiated rats treated with PL indicated a significant reduction in the level of MDA content and xanthine oxidase activity while there was an increase in the activity of xanthine dehydrogenase, SOD, catalase, and hepatic glutathione. Antioxidant activity of PL was not only induced by phenolic compounds but also by other compounds. For instance, secoiridoids and lignans were identified from PL and it was found that (+)-pinoresinol, (+)-medioresinol, (+)-pinoresinol-β-D-glucoside, and (−)-(7′S,8S,8′R)-4,4′-dihydroxy-3,3′,5,5′-tetramethoxy-7′,9-epoxylignan-9′-ol-7-one exhibited strong ABTS radical cation scavenging activity, while the compounds (+)-syringaresinol, (+)-isolariciresinol, and (+)-pinoresinol displayed significant ferric reducing antioxidant power (FRAP) [55]. The different antioxidant potentials of these compounds could be due to their structural differences (e.g., presence of phenolic hydroxyl groups) or the mechanism of action in different assays. In addition, Go and Song [22] suggested that the incorporation of 1% PL extracts into films (packaging materials) increased antioxidant activities that were measured using ABTS and DPPH radical scavenging assays.

### 7.2. Anticancer and Antitumor Effects

Persimmon leaves were reported to inhibit ROS elimination and cell proliferation. For example, ethanolic extract of PL, mainly containing flavonoids, was found to stimulate a platelet-derived growth factor receptor (PDGFR)-Ras-related C3 botulinum toxin substrate (Rac) signaling cascade in live cells [70]. They also found that downstream of the PDGFR-Rac pathway, where c-Jun N-terminal kinase (JNK) is activated by this extract. However, JNK-downstream inhibitors, including T-5224, cobalt chloride, and pepstatin A, attenuated PL-induced cell death. Moreover, Ding et al. [72] reported that flavonoids of PL showed higher cytotoxicity compared to the standard compounds (quercetin and rutin) in prostate cancer PC-3 cells. PL also induced PC-3 cell apoptosis by activation of oxidative stress, as revealed by MDA, ROS, nitrite, and iNOS activities, as well as mitochondrial-related apoptosis. In another study, PL extract (PLE), mainly containing flavonols with the 2″-galloyl moieties, was investigated on cellular DNA damage checkpoint signaling to sensitize cancer chemotherapy and it was found that PLE significantly improved the cytotoxicity of doxorubicin (DOX) in A549 lung adenocarcinoma cells [86]. PLE also reduced the phosphorylation of ataxia telangiectasia mutated (ATM) kinase in a dose-dependent manner. Moreover, Jeong et al. [59] reported NO scavenging and SOD-like activity of phenolic compounds of PL as well as their protective effects against ultraviolet B (UVB)-induced keratinocyte injury in HaCaT cells.

Apart from phenolics, polysaccharides of PL also exhibit potential anticancer and antitumor activities. For instance, polysaccharides obtained from PL were investigated for early metastasis of lung cancer and it was found that they suppressed transforming growth factor-beta 1 (TGF-β1)-induced epithelial-to-mesenchymal transition in A549 cells [73]. Park et al. [11] suggested that pectic polysaccharides of PL improved IL-12 and IL-6 production in peritoneal macrophages and induced natural killer cell-mediated tumoricidal effect and restrained tumor metastasis in mice in a dose-dependent manner. Similarly, pectic polysaccharides of PL inhibited tumor cell angiogenesis via vascular endothelial growth factor (VEGF) and matrix metalloproteinase (MMP-9) regulation [51].

### 7.3. Beneficial Effects on Eye-Related Diseases

Persimmon leaf compounds, mainly flavonoids and terpenoids, exhibit therapeutic potential against various eye-related diseases such as glaucoma, corneal neovascularization, dry eye disease, age-related macular degeneration, and edema. For example, Afzal and Hwang [63] treated human corneal endothelial cells (HCECs) with ethanolic extract of PL (EPL) and examined its impact on HCECs survival and Na^+^/K^+^-ATPase against cytotoxic drugs, namely ouabain (OU) and staurosporine (ST). Use of EPL restored viability of HCECs and enhanced Na^+^/K^+^-ATPase enzymatic activity with/without OU and ST; thus, EPL can be an effective material for corneal decompensation. Likewise, EPL showed potent protective effects on glutamate/1-buthionine-(S,R)-sulfoximine (BSO)-induced retinal ganglion cell (RGC)-5 death in vitro and protected RGCs from partial optic nerve crush (PONC)-induced retinal degeneration in vivo, thus suggesting its potential as an effective agent for preventing and treating glaucoma [64]. Similarly, EPL with its rich content of catechin, kaempferol, and quercetin showed potential for lowering intraocular pressure (IOP) with notable RGCs/optic nerve protection against retinal degeneration in a glaucoma mouse model [65]. In another study, EPL, mainly containing quercetin-3-*O*-β-glucoside, quercetin-3-*O*-β-galactoside, and quercetin-3-*O*-β-2″galloylglucoside, was used to examine N-methyl-N-nitrosourea (MNU)-induced retinal degeneration in mice and it was found that it significantly increased the retinal layer thicknesses, thus suggesting its potential use for the treatment of retinitis pigmentosa and age-related macular degeneration [31]. Recently, Peng et al. [35] identified six flavonoids, namely quercetin-3-*O*-β-galactoside, quercetin-3-(2-galloylglucoside), quercetin-3-*O*-β-glucoside, kaempferol-3-*O*-β-galactoside, kaempferol-3-(2-galloylglucoside), and kaempferol-3-*O*-β-glucoside, in rat eyes after oral administration of EPL. Therefore, EPL, mainly containing flavonoids, could serve as effective therapeutic agents for preventing and treating eye-related diseases.

### 7.4. Antidiabetic Effects

Persimmon leaves have demonstrated potential hypoglycemic effects, mainly by inhibiting/controlling α-amylase and α-glucosidase enzymes. For example, Hong et al. [66] suggested that Korean PL showed α-amylase and α-glucosidase inhibitory activities, possibly due to the presence of phenolics in the leaves. Similarly, PL compounds, mainly proanthocyanidins, decreased blood glucose levels in Wistar rats in a dose-dependent manner, which could be linked to the inhibition of pancreatic α-amylase [33]. Moreover, aqueous extract of PL inhibited α-glucosidase activity and enhanced glucose tolerance, improved blood lipid parameters, reduced body weight gain, suppressed fat accumulation in the liver, and maintained islet structure in *db*/*db* mice [41]. In addition, the novel glucosidase inhibitor, vomifoliol 9-*O*-α-arabinofuranosyl (1→6)-β-D-glucopyranoside, isolated from PL exhibited strong α-glucosidase inhibitory activity in HepG2 cells, suggesting that the PL could augment peripheral glucose as an insulin-sensitizing agent against Type 2 diabetes mellitus. In addition, addition of PL powder (5%, *w*/*w*) to a normal diet of C57BL/KsJ-*db*/*db* mice for 5 weeks decreased homeostatic index of insulin resistance (HOMA-IR), blood glucose, total cholesterol, and plasma triacylglycerol (TAG) levels, and also hepatic lipid droplets and liver weight, while increasing adiponectin and plasma high-density lipoprotein cholesterol (HDL-C) levels [69]. The anti-hyperglycemic potential was associated with the decreased effect of gluconeogenic enzymes and increased glucokinase activity and glycogen content in the liver, suggesting the potential for PL activity against type 2 diabetes. Furthermore, Khan et al. [7] developed a mass spectrometry (MS)-based proteomic approach to determine proteomic molecular signatures that could be applied to investigate the therapeutic potential of PL amelioration of diabetes. It was found that the salivary proteomic profile was changed after incorporating PL extract in prediabetic subjects, which could be used as potential protein signature candidates.

### 7.5. Antihyperlipidemic and Anti-Obesity Effects

Hyperlipidemia is linked with changes in lipid peroxide and lipid peroxidation. Persimmon leaves have been reported to improve lipid profiles and suppress body weight gain. For example, the hypolipidemic effects of powdered PL were investigated in rats fed on a high-fat diet (HFD) and it was found that PL significantly lowered the plasma TAG and total cholesterol (TC) contents, whereas it elevated the ratio of HDL-C/total-C and enhanced the atherogenic index [8]. Moreover, PL rich in phenolic compounds increased the hepatic 3-hydroxy-3-methylglutaryl (HMG)-CoA reductase and acyl-CoA:cholesterol acyltransferase (ACAT) activities, and also the contents of fecal TAG, cholesterol, and acidic sterol than the control groups. Similarly, flavonoids obtained from PL significantly decreased TC, TAG, and LDL-C, but increased HDL-C, lipoprotein lipase (LPL), and hepatic lipase (HL) in the lipid metabolic disorder of hyperlipidemia rats, indicating its lipid-lowering effect [75]. Similarly, Jung et al. [74] demonstrated the potential anti-obesity and lipid-lowering effects of persimmon leaves as they lowered body fats and improved plasma and hepatic lipid profiles in HFD-fed rats.

### 7.6. Immunostimulatory Effects

Immunostimulants are considered one of the body’s defense strategies for preventing and fighting against inflammatory diseases, infections, and cancer, among others. The bioactive compounds, mainly polysaccharides, of PL have been characterized for their immunostimulatory activities. The immunostimulatory potential of polysaccharides isolated from PL was investigated in RAW 264.7 macrophages, and their in vivo effects on cyclophosphamide-induced immunosuppression in mice were also examined [53]. The immunostimulatory effect of PL in interferon-γ (IFN-γ)-primed RAW 264.7 macrophages was noted as they enhanced the phagocytic effects and the expressions of immune modulators, including cytokines and inducible nitric oxide synthase (iNOS). PL increased the mRNA expressions of tumor necrosis factor-α (TNF-α), IL-6, and interleukin-1β (IL-1β). Similarly, pectic polysaccharides of PL phosphorylated mitogen-activated protein kinases (MAPK) and nuclear factor kappa-light-chain-enhancer of activated B cells (NF-κB) in RAW 264.7 cells in a dose-dependent manner [76]. Moreover, the effect of polysaccharides on IL-6 generation was completely suppressed by specific inhibitors of c-Jun N-terminal kinases (JNK) and extracellular signal-regulated kinases (ERK), suggesting macrophage activation. In another study, Shin et al. [54] suggested that the major polysaccharides in PL were 2-*O*-methyl-xylose, 2-*O*-methyl-fucoseapiose, 3-deoxy-D-*manno*-2-octulosonic acid, apiose, uronic acid, and 3-deoxy-D-lyxo-2-heptulosaric acid, which showed immune-stimulating activity. Meanwhile, triterpenoids of PL were reported to suppress tyrosyl phosphorylation of neutrophil proteins by inhibition of protein tyrosine kinase and stimulus-induced superoxide generation [46].

### 7.7. Neuroprotective Effects

Oxidative stress induced by ROS is linked to the pathogenesis of chronic neurodegenerative diseases, and PL, mainly their phenolic compounds, are reported to have potential neuroprotective effects in attenuating oxidative injury. For example, flavonoids isolated from PL were investigated in H_2_O_2_-induced apoptosis-like damage of NG108-15 cells and it was found that PL were able to protect the nerve cells from injury and apoptosis by upregulating Bcl-2 expression and increasing redox imbalance, thus suggesting the potential of PL in preventing and treating ischemia/reperfusion injury and other neurodegenerative diseases [17]. Quercetin and kaempferol, along with rutin, astragalin, hyperin, and isoquercitrin, were the main flavonoids in PL, which may play critical roles in attenuating oxidative injury. Similarly, Bei et al. [40] stated that flavonoids extracted from PL significantly protected rats from middle cerebral artery occlusion (MCAO) and four-vessel occlusion (4-VO) from ischemic injury in vivo and protected hippocampal neurons from glutamate-induced excitotoxic injury, indicating the potential neuroprotective effects of flavonoids (quercetin and kaempferol) due to their antioxidant activities. Furthermore, ethyl acetate extract of PL, rich in flavonoids, and triterpenoids exhibited potential protective effects on cognitive deficits induced by A*β* in rats, suggesting the possible inhibitory effect against Alzheimer’s disease. Apart from flavonoids, secoiridoids, and lignans isolated from PL also demonstrated potential neuroprotective effects. For instance, secoiridoids and lignans, mainly (+)-medioresinol and (+)-syringaresinol-β-D-glucoside, showed potential neuroprotective effects against neuroblastoma SH-SY5Y cell injury induced by H_2_O_2_ [55].

### 7.8. Antiallergic, Antiwrinkle, Anti-Inflammatory, Anti-Tyrosinase, and Antibacterial Effects

Persimmon fruits have been used as key components in a few marketed cosmetic products, namely deodorizing and purifying body lotion, soaps, skin toners, body wash, and body serums [6]. Therefore, much attention has been paid to PL as they contain anti-inflammatory, antiallergic, antiwrinkle, photo-protective, anti-tyrosinase, antioxidant, and antibacterial substances. These include flavonoids (e.g., tannins, astragalin, isoquercitrin, and catechins) and terpenoids (rotungenic, ursolic, and oleanolic acids), exhibiting the potential to inhibit tyrosinase, elastase, and collagenase enzymes. For example, Kotani et al. [83] examined the inhibitory effect of PL extracts, mainly containing flavonoids (astragalin), on the histamine release by KU812 cells and found inhibitory effects on the atopic dermatitis model mice. Moreover, an ethanolic extract of PL rich in flavonoids exhibited xanthine oxidase, tyrosinase, and elastase inhibition, suggesting the potential of PL as an antiwrinkle agent [81]. Moreover, Kashif et al. [6] summarized the antiallergic properties of PL, mainly phenolic compounds, and their potential use in contact dermatitis. Yoo et al. [80] suggested that PL extracts rich in phenolics inhibited the cutaneous anaphylaxis reaction activated by anti-dinitrophenyl immunoglobulin E (IgE) antibody in mice and NO production in lipopolysaccharide (LPS)-stimulated RAW 264.7 cells, thus suggesting their potential for prevention and treatment of allergy-related diseases. Furthermore, triterpenoids (coussaric and betulinic acids) obtained from PL demonstrated anti-inflammatory activities via inhibition of NF-kB pathway [87]. Furthermore, they also inhibited prostaglandin E2 (PGE2) and NO production in LPS-activated RAW 264.7 cells, suppressed interleukin-6 (IL-6), TNF-α, and IL-1β levels, and decreased protein expression of iNOS and cyclooxygenase-2. Similarly, water-soluble extracts obtained from PL were a rich source of phenolic compounds, which suppressed the production of inflammatory mediators (NO and PGE2) and pro-inflammatory cytokines (TNF-α and IL-1β) in LPS-stimulated RAW 264.7 macrophages [68].

In addition, Cho et al. [39] reported the potential of PL as a cosmetical ingredient with a potent whitening effect due to the ability of phenolic compounds (e.g., (+)-gallocatechin and prodelphinidin B-3) obtained from PL to show inhibition against melanin biosynthesis in melanoma cells and tyrosine enzymes. Tyrosinase is involved in the biosynthesis of melanin pigments and excessive melanin is linked to skin darkening and neurodegenerative disorders [29]. In another study, Xue et al. [34] identified different phenolic compounds from PL and found that only chrysontemin showed tyrosinase inhibitory activity, and also suggested that the most prominent moiety in inhibiting tyrosinase enzyme was the 3′,4′-dihydroxy groups of the catechol. The inhibitory mechanism of polyphenols could be related to their chelation ability toward binuclear copper. Furthermore, triterpenoids, mainly pomolic, ursolic, and oleanonic acids, with a 3β-hydroxy or a 3-carbonyl group isolated from Korean PL were reported to have protein tyrosine phosphatase 1B inhibitory activity, while those with a 3α-hydroxy moiety were not effective [50]. Moreover, PL have shown antibacterial activity against food-borne pathogens (e.g., *Escherichia coli, Listeria monocytogenes, Salmonella typhimurium, Fluorescence pseudomonas, Staphylococcus aureus, Bacillus cereus, Bacillus subtilis*, and *Proteus vulgaris*) and the active compounds were recognized as flavonoids, coumarins, volatile oils, and organic acids [10,23,66]. Arakawa et al. [88] reported the antibacterial activity against *E. coli* and *S. aureus* as well as *Streptococcus mutans*, *Campylobacter sputorum*, and *Bacteroides thetaiotaomicron*; this function could be related to the presence of tannins in PL.

### 7.9. Other Effects

Angiotensin-converting enzyme (ACE) inhibitors prevent the production of angiotensin II in the body, a substance that narrows blood vessels, maintaining lower blood pressure. PL have been found to have ACE inhibitory activity, possibly due to the presence of phenolic compounds, including proanthocyanidins [23,30,66]. Kawakami et al. [67] applied proanthocyanidins of PL to hypertensive rats, rat aortas, and human umbilical vein endothelial cells to investigate the antihypertensive effect and found that proanthocyanidins significantly decreased the systolic blood pressure. The phosphorylation contents of endothelial NO synthase (Ser-1177) and the upstream kinase Akt (Ser-473) in umbilical cells were also improved, suggesting the antihypertensive effect of PL via endothelium-dependent NO/cyclic guanosine monophosphate (cGMP) pathway.

The polysaccharide fraction of PL was collected after purification by gel filtration (G-100 and G-150), as well as fast protein liquid and phenyl superpose column chromatography, which showed anticoagulant activity by inhibiting thrombin via binding to fibrinogen binding sites [77]. Moreover, anti-osteoporotic effects of polysaccharides (e.g., galacturonic acid, galactose, and arabinose) isolated from PL were investigated using an in vitro system of receptor activator of nuclear factor-κB ligand (RANKL)-induced osteoclast differentiation and in vivo model of ovariectomy (OVX)-induced bone loss. PL significantly decreased OVX-induced trabecular bone loss by suppressing osteoclast activity and also inhibited osteoclast differentiation. In addition, Yu et al. [79] suggested that PL showed antidepressant-like effects, indicating that PL could be considered a potential candidate as an antidepressant. This is because PL extract improved sucrose preference and decreased immobility times in the forced swim test (FST) and tail suspension test (TST), but did not affect locomotor activity, for the treatment of chronic social defeat stress (CSDS)-subjected mice. Moreover, PL extract increased synaptic protein levels, cyclic adenosine monophosphate (cAMP), brain levels of platelet serotonin (5-HT), brain-derived neurotrophic factor (BDNF), postsynaptic density synapsin-1 and protein 95 (PSD95), phosphorylated (p)-cAMP-response element binding protein (CREB), and synapsin-1; it also inhibited dendritic spine loss in mice.

## 8. Bioavailability of PL

The bioavailability of many phenolic compounds, especially flavonoids, found in PL is relatively low due to their poor solubility. In order to enhance their oral bioavailability, Zhang et al. [78] prepared phospholipid complexes of flavonoids (quercetin and kaempferol) from PL (PCF-PL) and monitored their anti-atherosclerotic properties in atherosclerotic rats. The relative bioavailabilities of kaempferol and quercetin in PCF-PL compared to PL were 337 and 242%, respectively. Based on the morphological changes of the aorta and the levels of biochemical parameters in serum, PCF-PL showed better therapeutic potential in the treatment of atherosclerotic disease than PL. Similarly, a nano-emulsifying drug delivery system (NEDDS) was developed from PL (kaempferol and quercetin) and compared with commercially available tablets (NXQ) [89]. Compared with the NXQ, the AUC (area under the concentration–time curve) of kaempferol and quercetin isolated from PL was increased 1.6- and 1.5-fold, respectively, in fasting beagle dogs. Thus, the bioavailability of quercetin and kaempferol was significantly enhanced by nano-emulsifying, possibly due to increased drug concentration in the GI tract and absorption area. Flavonoids of PL, such as flavonoid glycosides, could be metabolized to flavonoid aglycones by the action of microbiota after administering, followed by the absorption in the intestinal tract in the aglycone form and then glycosylated after being absorbed in the blood and found again in glycosylated form in the plasma [90]. For example, the microbial metabolism of flavonoids, isolated from PL, by intestinal bacteria was investigated in vitro; the contents of flavonoid aglycones and flavonoid glycosides was immediately decreased by intestinal bacteria [90]. Moreover, after oral administration of PL flavonoids, in vivo metabolites were detected in rat plasma and urine. Eight and three flavonoids were identified in the urine and plasma, respectively, using high-performance liquid chromatography–linear trap quadrupole orbitrap–mass spectrometry, but no flavonoid aglycones were detected in the plasma. In addition, Heras et al. [37] suggested that the bioavailability and total antioxidant capacity (TAC) in the aqueous extract of PL was lower than that in the fruit of persimmon or its fibers, possibly due to the lower content of antioxidants in fruit/fibers.

## 9. Conclusions

Persimmon leaves have gained much interest from functional food, nutraceutical, pharmaceutical, and cosmeceutical industries due to their impressive nutritional and bioactive profiles. However, better understanding and unravelling of the detailed bioactive compounds, and their isolation, characterization, structure identification, and determination of their biological activities is required; these include use of appropriate models and clinical approaches. Moreover, the bioaccessibility and bioavailability of these biomolecules and their detailed mechanisms of action need to be established in order to support the health claims from traditional practices. In addition, in-depth studies should be conducted related to PL-based product development and physicochemical properties, sensory characteristics, and microbial stability.

## Figures and Tables

**Table 1:** Major phenolic compounds in persimmon leaves.

Species/Cultivars	Origin	TPC (mg GAE/g)	TFC (mg CE/g)	TTC (mg CE/g)	Individual Compounds (µg/g)	References
*D. kaki* (Sangju dungsi)	Korea	90.41	30.67	47	NA	[13]
*D. kaki* (Sangju-dungsi, Sangamdungsi, Gabjubaekmok, Cheongdobansi, and Suhong)	Korea	72.59–112.09	30.27–37.83	28.67–81.33	NA	[4]
*D. lotus* (Dongsi)	Korea	58.01–58.16	14.16–15.83	32.38–35.46	NA	[24]
*D. kaki*	Korea	NA	NA	NA	Catechin, gallocatechin, pyrocyanidin C-1, procyanidin B-1, prodelphinidin B-3, procyanidin B-7-3-*O*-gallate, gallocatechin-(4*α*→8)-catechin, procyanidin C-1-3′-3″-3″-*O*-trigallate, and epigallocatechin-(4*β*→8)-epigallocatechin-(4*β*→8)-catechin	[30]
*D. kaki*	Korea	NA	NA	NA	Quercetin, quercetin-3-*O*-β-glucoside, quercetin-3-*O*-β-galactoside, quercetin-3-*O*-β-2″-galloylglucoside, kaempferol, kaempferol-3-*O*-β-glucoside, kaempferol-3-*O*-β-galactoside, and kaempferol-3-*O*-β-2″-galloylglucoside	[31]
*D. kaki*	Korea	NA	NA	NA	Isoquercetin, quercetin 3-*O*-β-D-glucopyranoside-2″-gallate, kaempferol 3-*O*-β-D-glucopyranoside-2″-gallate, and astragalin	[32]
*D. kaki*	Korea	NA	NA	NA	Catechin, hyperoside, quercetin, isoquercitrin, trifolin, astragalin, quercetin-3-*O*-β-2″-galloylgalactoside, quercetin-3-*O*-β-2″-galloylglucoside, kaempferol, kaempferol-3-*O*-β-D-2″-coumaroylgalactoside, kaempferol-3-*O*-β-2″-galloylgalactoside, kaempferol-3-*O*-α-arabinoside, and scopoletin	[16]
*D. kaki* (Hiratanenashi and Tonewase)	Japan	26–27.7	NA	NA	Proanthocyanidins (catechin, epigallocatechin, epigallocatechin-3-*O*-gallate, epicatechin, epicatechin-3-*O*-gallate, and prodelphinidin)	[33]
*D. kaki*	Japan	112	58.4	NA	NA	[15]
*D. kaki* (Fuyu, Jiro, Kinsyu, Tanrei, Yotsumizo, and Saijo)	Japan	NA	NA	NA	Isoquercitrin, hyperoside, trifolin, chrysontemin, astragalin, kaempferol-3-*O*-(2″-*O*-galloyl-β-D-glucopyranoside), and quercetin-3-*O*-(2″-*O*-galloyl-β-D-glucopyranoside)	[34]
*D. kaki*	China	NA	NA	NA	Quercetin-3-*O*-β-glucoside, quercetin-3-*O*-β-galactoside, quercetin-3-(2-galloylglucoside), kaempferol-3-*O*-β-glucoside, kaempferol-3-*O*-β-galactoside, and kaempferol-3-(2-galloylglucoside)	[35]
*D. kaki* (Tonewase, Fuyu, Aoso, Hachiya, Diamond Bull Heart, and Bull Heart)	Taiwan	69.27–149.59	40.78–90.62	12.58–19.23	Protocatechuic acid, gallic acid, *p*-hydroxybenzoic acid, vanillic acid, chlorogenic acid, caffeic acid, *p*-coumaric acid, sinapic acid, catechin, epicatechin, myricetin-3-*O*-glucoside, myricetin-3-*O*-rhamnoside, rutin, quercetin-3-*O*-glucoside, quercetin-3-*O*-galactoside, quercitrin, quercetin-3-*O*-arabinoside, kaempferol-3-*O*-rutinoside, kaempferol-3-*O*-glucoside, myricetin, naringin, kaempferol-3-*O*-rhamnoside, isorhamnetin-3-*O*-rutinoside, naringenin-7-*O*-glucoside, isorhamnetin-3-*O*-glucoside, quercetin, kamempferol, apigenin, and isorhamnetin.	[36]
*D. kaki* (Rojo brillante)	Spain	86	22.9		NA	[37]
*D. kaki* (Rojo brillante)	Spain				Gallic acid-*O*-hexoside (10.3), gallic acid (32.5), gallocatechin (442.2), catechin-*O*-hexoside I (19), procyanidin B1 (203.71), procyanidin dimer I (54.6), catechin (435.2), procyanidin dimer II (22.6), prodelphinidin dimer B3 (24.4), myricetin-*O*-hexoside I (304.8), myricetin-*O*-hexoside II (563.4), isoquercetin (247.4), quercetin-*O*-hexoside (348.8), quercetin-*O*-pentoside I (31.9), quercetin-*O*-pentoside II (52), kaempferol-3-*O*-glucoside (165.3), kaempferol-*O*-hexoside I (176.8), myricetin (44.7), quercetin (354.7), kaempferol (206.2), and isorhamnetin (42.8)	[3]
*D. kaki*	China				Astragalin, trifolin, annulatin, myricetin, myricetin-3-*O*-glucopyranoside, quercetin, vitexin, hyperoside, quercetin-3-*O*-galloylglucoside, isorhamnetin-3-β-D-glucopyranoside, isorhamnetin-3-β-D-galactoside, kaempferol, kaempferol-3-*O*-galloylglucoside, kaempferol-3-*O*-galloylgalactoside, and salvianolicacid D	[5]

TPC, total phenolic content; TFC, total flavonoid content; TTC, total tannin content; GAE, gallic acid equivalents; and CE, catechin equivalents.

**Table 2:** Health-promoting properties and mechanisms of actions demonstrated by PL.

Health Effects	Species/Cultivars	Origin	Responsible Compounds/Extracts	Results/Mechanisms	References
Beneficial actions against eye-related diseases	*Diospyros kaki*	Korea	Ethanolic extracts (flavonoids)	Showed the potential to be an effective agent against corneal edema and related corneal disorders	[63]
	*D. kaki*	Korea	Ethanolic extracts	Exhibited protective properties against retinal degeneration (e.g., glaucoma) in vitro and in vivo	[64]
	*D. kaki*	Korea	Ethanolic extracts (catechin, kaempferol, and quercetin)	Reduced elevated intraocular pressure in mouse models of glaucoma	[65]
	*D. kaki*	Korea	Ethanolic extracts (quercetin)	Showed the potential to prevent degenerative retinal diseases (retinitis pigmentosa and age-related macular degeneration)	[31]
	*D. kaki*	China	Ethanolic extracts (flavonoids)	Potential effect in lowering the degeneration of retina	[35]
Antihypertensive	*D. kaki*	Korea	Prodelphinidin B-3, procyanidin B-7-3-*O*-gallate, procyanidin C-1-3′-3″-3″-*O*-trigallate, and epigallocatechin-(4β→8)-epigallocatechin-(4β→8)-catechin)	Showed angiotensin-converting enzyme (ACE), xanthine oxidase, and tyrosinase inhibitory activities	[30]
	*D. kaki*	Korea	Extracts	Showed ACE inhibitory activity	[23,30,66]
	*D. kaki*	Japan	Proanthocyanidins	Showed activity via an endothelium-dependent nitric oxide/cGMP pathway	[67]
Anti-inflammatory	*D. kaki*	Korea	Water extracts	Suppressed the production of inflammatory mediators and pro-inflammatory cytokines	[68]
Neuroprotective	*D. kaki*	China	Flavonoid extracts	Showed the potential to prevent and treat ischemia/reperfusion injury and other related neurodegenerative diseases	[17,40]
	*D. kaki*	China	Secoiridoids and lignans	Showed potential neuroprotective activity	[55]
Antidiabetic	*D. kaki*	Korea	Aqueous extracts	Exhibited activity via α-glucosidase inhibition and maintenance of functional β-cells	[41]
	*D. kaki*	Korea	Extracts (quercetin 3-*O*-2″galloylglucoside and kaempferol 3-*O*-2″galloylglucoside)	Showed therapeutic potentials in diabetes amelioration	[7]
	*D. kaki*	Korea	Methanolic extracts	Showed α-glucosidase and α-amylase inhibition	[66]
	*D. kaki*	Korea	PL powder enriched with phenolic compounds	Improved hyperglycemia by alterations in activity and/or mRNA expression of hepatic enzymes linked in glucose utilization and production	[69]
	*D. kaki*	Japan	Proanthocyanidins (mainly epigallocatechin-3-*O*-gallate)	Inhibited α-amylase and decreased blood glucose level in Wistar rats	[33]
	*D. kaki*	Korea	Vomifoliol 9-*O*-α-arabinofuranosyl (1→6)-β-D-glucopyranoside	Stimulated the glucose uptake in HepG2 and 3T3-L1 cells	[43]
Anti-tyrosinase	*D. kaki*	Japan	Chrysontemin	Exhibited activity against tyrosinase for oxidation of levodopa	[34]
	*D. kaki*	Korea	Ethanolic extracts (prodelphinidin B-3 and (+)-gallocatechin)	Showed tyrosinase inhibitory activity	[39]
	*D. kaki*	Korea	Triterpenoids	Inhibited protein tyrosine phosphatase 1B activity	[50]
Anticancer and antitumor	*D. kaki*	Korea	Ethanolic extracts (mainly quercetin and kaempferol)	Triggered PDGFR-Rac-JNK signaling cascade in live cells, causing cancer cell death	[70]
	*D. kaki*	China	Flavonoids	Decreased the level of reactive oxygen species (ROS) and malondialdehyde (MDA) in MC3T3-E1 cells	[71]
	*D. kaki*	China	Flavonoids	Reduced H_2_O_2_-induced apoptosis in MC3T3-E1 cells via the NF-kB pathway	[9]
	*D. kaki*	China	Flavonoids	Induced apoptosis in PC-3 cells by activation of oxidative stress and mitochondrial apoptosis	[72]
	*D. kaki*	Korea	Phenolic compounds	Exhibited protective effect against ultraviolet B (UVB)-induced cell cytotoxicity	[59]
	*D. kaki*	Korea	Pectic polysaccharides (mainly acidic sugars, rhamnose, arabinose, and galactose)	Inhibited vascular endothelial growth factor and matrix metalloproteinase (MMP-9) expression in human umbilical vein endothelial cells via regulation of PI3K/AKT, p38, JNK, and NF-kB p65 signaling pathways	[51]
	*D. kaki*	Korea	Pectic polysaccharides	Increased levels of IL-6 and IL-12 produced by peritoneal macrophages	[11]
	*D. kaki*	Korea	Polysaccharides	Suppressed TGF-β1-induced epithelial-to-mesenchymal transition in A549 cells	[73]
	*D. kaki*	Korea	Polysaccharides (mainly neutral sugars and uronic acid)	Up-regulated the expressions of iNOS, TNF-α, IL-1β, and IL-6 genes by activating TLR2-mediated NF-kB activations	[53]
Antihyperlipidemic and anti-obesity	*D. kaki*	Korea	PL extracts	Lowered body fat weight and improved plasma and hepatic lipid profiles in high-fat diet (HFD)-fed rats	[74]
	*D. kaki*	Korea	PL powder enriched with phenolic compounds	Improved plasma and hepatic lipid levels profile via the increased fecal lipids in HFD rats	[8]
	*D. kaki*	China	Flavonoids	Improved lipid metabolic disorder in hyperlipidemic rats	[75]
Immunostimulatory	*D. kaki*	Korea	Pectic polysaccharides (neutral sugars and uronic acid)	Stimulated the immune activity (IL-6/IL-12 and TNF-α production) of peritoneal macrophages cells	[76]
	*D. kaki*	Korea	Polysaccharides	Exhibited immuno-stimulating activity	[54]
	*D. kaki*	Japan	Triterpenoids	Induced superoxide generation and tyrosyl phosphorylation in human polymorphonuclear leukocytes	[47]
Anti Alzheimer’s	*D. kaki*	China	Ethyl acetate extract (flavonoids and triterpenoids)	Showed a potent protective effect on cognitive deficits induced by A*β* in rats	[5]
Anticoagulant	*D. kaki*	Korea	PL extracts	Delayed thrombin time (TT), activated partial thromboplastin time (APTT), and prothrombin time (PT) in human plasma	[77]
Anti-osteoporotic	*D. kaki*	Korea	Polysaccharides, mainly neutral sugars and uronic acid	Improved ovariectomy-induced trabecular bone loss by suppressing osteoclast activity	[52]
Anti-atherosclerotic	*D. kaki*	China	Phospholipid complexes flavonoids	Improved the bioavailability in vivo and anti-atherosclerotic properties in atherosclerosis rats	[78]
Antidepressant	*D. kaki*	China	PL extracts	Showed antidepressant-like effect in chronic social defeat stress-subjected mice and improved neurogenesis	[79]
Antiallergic and antiwrinkle	*D. kaki*	Korea	Ethanolic extracts (prodelphinidin B-3 and (+)-gallocatechin)	Showed inhibitory activity against tyrosinase and melanin biosynthesis in melanoma cell	[39]
	*D. kaki*	Korea	Phenolic extracts	Exhibited antiallergic effect	[80]
	*D. kaki*	Korea	Ethanolic extracts (flavonoids)	Showed xanthine oxidase, tyrosinase, and elastase inhibitory activities	[81]
	*D. kaki*	Korea	Ethyl acetate extracts	Inhibited xanthine oxidase	[82]
	*D. kaki*	Japan	Flavonoids (astragalin)	Inhibited histamine release from KU812 cell in response to cross-linkage of FcεRI (high-affinity IgE receptor)	[83]
Antimicrobial	*D. kaki*	Korea	Phenolic extracts	Showed inhibitory activity against *Listeria monocytogenes, Staphylococcus aureus, Escherichia coil*, *and Salmonella typhimurium*	[23]
	*D. kaki*	Korea	Methanolic extracts (polyphenols)	Exhibited inhibition against *E. coli* O157:H7	[66]

**Table 3:** Antioxidant activity of bioactive compounds extracted from PL.

Species/Cultivars	Origin	Responsible Compounds	DPPH RSA (%)	ABTS RSA (%)	Hydroxyl RSA (EC_50_ µg/mL)	TEAC (µmol TE/g)	Reducing Power (EC_50_ µg/mL)	References
*D. kaki*	Korea	Phenolic compounds	48.86	88.17	NA	NA	NA	[13]
*D. kaki*	Korea	Phenolic compounds	48.19–54.09	73.85–94.66	NA	NA	NA	[4]
*D. kaki*	Korea	Phenolic compounds	64.47 (IC_50_ µg/mL)	NA	NA	NA	NA	[66]
*D. lotus*	Korea	Phenolic compounds	26.5–27.22	75.24–75.37	NA	NA	NA	[24]
*D. kaki*	Spain	Phenolic compounds	NA	NA	NA	122 (mg TE/g)	NA	[37]
*D. kaki*	Spain	Phenolic compounds	105–190 (mg TE/g)	NA	NA	NA	NA	[2]
*D. kaki*	Taiwan	Phenolic compounds	56.74–98.84 (EC_50_ µg/mL)	NA	NA	647.14–951.1	278.86–441.41	[36]
*D. kaki*	China	Flavonoids	96.36 (EC_50_ µg/mL)	NA	111.23	NA	NA	[71]
*D. kaki*	China	Naoxinqing tablet (astragalin, isoquercitin, quercetin, kaempfero, and 3,4-dihydroxybenzoic acid)	119–181 (EC_50_ µM)	68–350 (EC_50_ µM)	NA	NA	NA	[42]
*D. kaki*	China	Secoiridoids and lignans	31.2–109.9 (IC_50_ µg/mL)	3.6–22.9 (IC_50_ µg/mL)	NA	NA	NA	[55]

RSA, radical scavenging activity; TEAC, Trolox equivalent antioxidant capacity; TE, Trolox equivalents; and NA, not available.

## Data Availability

Not applicable.
